# Supplementary material for: Are carbon emissions trading and green financial instruments synergistic? -Comprehensive quantitative research based on content analysis
Source: PLoS One. 2024 Mar 7;19(3):e0298601. doi: 10.1371/journal.pone.0298601 (PMC10919601; doi:10.1371/journal.pone.0298601)
Supplement: S1 Data — (ZIP) [file pone.0298601.s001.zip › Data/地级市Z_F工作报告.zip!/┴╔─■/╙¬┐┌╩╨/└┤╫╘╣1⁄2╓┌║┼í╛┬φ┐╦╩2╛▌═°í┐_2004.docx]

各位代表：

　　我受市人民政府的委托，向大会作政府工作报告，请予审议，并请政协委员和列席会议的同志提出意见。

　　一、２００３年工作的回顾

　　２００３年是贯彻党的十六大精神的第一年。一年来，在市委的正确领导下，我们认真贯彻“三个代表”重要思想，以深化改革、扩大开放、优化结构、项目开发和环境建设为重点，正确处理改革、发展、稳定的关系，努力克服“非典”带来的不利影响，实现了国民经济的快速增长和社会事业的全面发展。全年完成国内生产总值２５３．５亿元，比上年增长１６．２％。固定资产投资１０３．６亿元，增长４０．４％。财政一般预算收入１０．７亿元（新口径），增长２０．９％。城市人均可支配收入７３１８元，增长１３．４％。农民人均纯收入３５６１元，增长７．８％。

　　农村经济全面发展。全年完成第一产业增加值３２．７亿元，增长９％。实现农业总产值５６．５亿元，增长９．７％。农业结构进一步优化，粮经比例调整为５７?押４３，订单农业面积达到７０万亩。粮食总产量４７．１万吨。全年新栽果树３６９万株，水果总产量３８．７万吨，增长１４．６％。水产工厂化养殖面积达９万平方米，增长３４％；水产品总产量实现３２万吨，增长１２．３％。肉、蛋、奶产量均比上年有一定增长。完成人工造林１４．６万亩，植树３１００万株，退耕还林９万亩。积极推进农业产业化经营，建设了３０个龙头企业，农副产品加工转化率达到３２％。加快仙人岛等１０个现代农业园区建设，现已建成２８个功能区，入区项目达８０个。积极改善农业生产条件，新建灌溉工程４１９项，加固堤防１５０公里，治理水土流失面积１９．２万亩。乡镇企业继续保持良好的发展势头，全年投资３０．５亿元，新上百万元以上项目５３２项；乡镇企业实现增加值１３９．５亿元，增长１７．８％。

　　工业经济运行质量明显提高。全年完成第二产业增加值１３０．１亿元，增长２０．２％。规模以上工业总产值２７６．３亿元，增长２９．１％；实现利润９．７亿元，增长５６．８％。大力推进企业的机制创新、体制创新和技术创新，对４０户市直企业实施了改革，分流富余人员２．２万人，减轻债务负担２亿元，盘活存量资产４．５亿元，吸引资金８．３亿元。完成工业固定资产投资５０．５亿元，增长６９．３％；完成技术改造投资１１亿元，增长２０．１％。开发新产品５６０种，实现新产品产值３１亿元。全市已拥有省级以上名牌产品３２种，名牌产品产值占全市工业总产值的３０％。加大民营经济扶持力度，为民营企业新增贷款１４亿元，中小企业信用担保中心担保贷款６０００多万元。全年民营企业新上投资５００万元以上工业项目３０４个，占全市新上工业项目总数的８２．８％。规模以上民营企业达６０４户，占全市总量的８５．９％。实现民营经济增加值１５８亿元，增长１８．２％，占全市ＧＤＰ的６２．３％。

　　第三产业稳步增长。全年完成第三产业增加值９０．７亿元，增长１３．５％。围绕完善市场体系，开工建设各类市场１０处，北方陶瓷商城全年销售陶瓷、建材制品１３０万吨，进入全国大型市场行列。商品流通不断扩大，社会消费品零售总额达到８１．５亿元，增长１６．９％。大力兴办信息、咨询、广告、社区服务等新兴第三产业，强化了社会服务功能。旅游总收入实现１１．４亿元，下降１０％。加速发展保险、邮电通信业，全年保险费总收入６．９亿元，增长３５％；通信业务总量１３．９亿元，增长２１．５％。

　　开放型经济实现新突破。全年批准外商投资企业１１８家，外商直接投资２．４８亿美元，增长１５．３％。完成外贸进出口总额８．４１亿美元，增长２９％，其中出口６．０７亿美元，增长２２．４％，高于全省平均增幅４．１个百分点。派出和接待经贸友好团组１７０个、１３８５人次；境外劳务输出１９１６人，增长１２％。开发区新批及追加外资项目３６个，实现外商直接投资６５１５万美元，增长２７．６％；外贸出口２．４亿美元，增长１９．７％。高新区完成基础设施投资１５００万元，进区企业达２２户，实现技工贸总收入２．７３亿元，增长３４％。营口港完成固定资产投资１５．５亿元，航道改造、集装箱泊位、散粮转运设施等工程顺利完工。港口吞吐量完成４００９万吨，增长２８．２％；集装箱运量４０．２万标箱，增长３３．９％。

　　城乡基础设施不断改善。稳步推进城市资产市场化运作，全年出让土地使用权３０宗，面积９９．８万平方米，土地出让金总额达４．５亿元。建立了土地收购储备制度，已收购储备土地２２宗，面积８２万平方米。加强矿业市场培育，对６宗矿山开采权进行公开出让，出让金达８８３３．５万元。继续活跃房地产市场，全年实现交易额９８亿元，增长５５．６％。集中力量抓好重点工程建设，鲅鱼圈水源工程全部竣工，污水治理土建工程基本完成，市区供水管网扩建已开工。完成交通建设投资２．１亿元，农村公路网建设实现年计划的１８６％。年初确定的１５件实事有１３件完成或超计划完成，其余２项工程正在实施。积极推进公用事业和建筑企业改革，加强城市综合执法工作，市容市貌进一步改观，交通管理达到Ｃ类城市二级水平。大力实施“绿化工程”，完成城市植树１６０．１万株，新增公共绿地２１．８万平方米。加强环境保护工作，大气质量稳定在二级标准之内，城乡环境质量进一步优化。

　　财政金融运行平稳。积极培植和壮大财源，加强税收征管，全口径财政收入完成３２．８亿元，增长２３．４％。继续深化财政体制改革，进一步完善部门综合预算、会计集中核算和国库集中支付制度，有效地调整了财政支出结构，确保了重点需要。积极稳妥地推进农村税费改革，按期完成第一阶段任务，全市农民总体减负１．６亿元，减负率为７４．７％。努力扩大城乡储蓄，各项存款余额达到２８８．７亿元，增长１４．８％；各项贷款余额２５２．６亿元，增长１６．９％。不断开辟新的融资渠道，吸引浦发、光大、广发等股份制商业银行进入我市金融市场。争取国家专项借款１０亿元，使多年形成的财政证券风险得以化解。努力提高信贷质量，严厉打击逃废债行为，不良贷款率比年初降低９．７个百分点，金融环境进一步改善。

　　“项目年”活动取得可喜成果。以“项目年”工作统揽全局，全市上下形成了千军万马跑项目、群策群力上项目的可喜局面。坚持政策调动和服务推动，制订出台了一系列优惠政策，建立了３个项目服务中心，实行“一站式”办公、“一条龙”服务，营造了宽松的发展环境。规划建设了１２个工业园区，累计进区企业５９１户，其中规模以上企业１７０户。全年开工建设（含续建）投资５００万元以上项目５１２项，新增规模以上工业企业１１０户，全面完成了“项目年”所确定的目标。

　　社会保障能力进一步增强。积极开辟就业渠道，全年新增就业６８７１３人次，城镇登记失业率为５．８％，低于省控制目标０．２个百分点。全市参加企业基本养老保险的职工达到２５．８万人，新增参保人数２万人；失业保险参保人数２４．８万人，完成省参保计划的１１０％；医疗保险参保人数２４．２万人，覆盖面为７１％，完成省扩面任务的２．１倍。对低保实行动态管理，享受最低生活保障的人数稳定在６．３万人，累计发放保障金７７４４万元。各街道、社区已全部建立劳动和社会保障工作机构，市内８８个社区整合为５１个，精简比例达４２％。全年实现并轨２６３０２人，完成年计划的１０５％。深入开展扶贫帮困活动，筹集并安排扶贫帮困资金和物资折款２７００多万元，救助１３万人次。

　　营口老工业基地振兴起步扎实。按照省委、省政府要求，结合我市实际，就经济结构调整、深化企业改革、扩大对外开放等１３个专题进行了深入调研，在此基础上形成了《营口市老工业基地调整改造振兴规划》。积极争取国家、省有关部门的支持，实现在产业发展重点和重大项目安排上与国家、省总体规划相衔接，目前已有一批项目得到国家和省的支持，其中有３个项目列入东北等老工业基地振兴国债贴息项目计划。

　　各项社会事业协调发展。积极推进科技进步，全年签订长期科技合作协议９２项，完成专利申请４０３件。高新技术企业达到３２家，有２７个高新技术产业化项目批量投产，实现产值４２亿元，增长２７．２％。建设和完善农业科技示范基地１３个，引进开发农业新品种４２个。我市被国家科技部批准为镁质材料产业化基地，并成功举办了中国镁质材料新技术新产品专项对接会，签订合同３６项。大力发展教育事业，以市（县）、区为主的农村教育管理体制改革不断深化，制定实施了“双高”“普九”规划，加大了“控辍”力度，圆满完成了农村中小学校布局调整三年规划任务。文化基础设施不断完善，群众文化活动广泛开展，文化市场进一步净化和繁荣。广播电视事业有了新的发展，有线电视网络和宽带数据网络覆盖面不断扩大，节目质量有所提高。计划生育工作得到加强，人口自然增长率控制在１．３８‰。体育工作取得新进展，群众性体育活动蓬勃开展，竞技体育水平不断提高。

　　民主和法制建设得到加强。各级政府自觉接受人大及其常委会依法监督和政协民主监督，办理人大代表建议和政协提案４９１件，办复率１００％，满意率９５％以上，解决率７９．６％。加强与民主党派、工商联、人民团体及各界人士的联系，提高了政府决策的民主化、科学化水平。认真贯彻落实党的民族、宗教和侨务政策，有效地解决了一些难点问题。深入开展“四五”普法活动，认真落实行政执法责任制，增强了全民的法律意识，提高了依法行政水平。切实加强社会治安综合治理，严厉打击各种刑事犯罪和“法轮功”等邪教组织活动，全年破获各类刑事案件５８６１起。继续开展“扫黄”、“打非”、禁毒等专项斗争，进一步净化了社会环境。积极推进警务改革，公安队伍素质有新的提高。认真做好信访工作，按照“分级负责，归口办理”的原则，建立了领导包案、责任追究等项工作制度，维护了社会稳定。国防教育和民兵、预备役工作得到加强，我市进入全国“双拥”模范城行列。

　　“防非”工作取得阶段性胜利。面对突如其来的“非典”疫情，按照国家和省统一部署，采取强化领导、全民动员、建立网络、把住入口、查清内部、突出重点、科学防治等措施，确保全地区“非典”诊断和疑似病例为零，维护了全市的生产、工作、学习和社会秩序。以“防非”为契机，积极开展农村卫生市场清理整顿活动，促进了城乡卫生体系和医疗保健体系建设。

　　各位代表，过去的一年，是全市上下通力协作、奋力拼搏、开拓进取的一年，也是我市改革开放、经济建设和各项事业取得丰硕成果的一年。这些成绩的取得，是在市委正确领导下，在人大依法监督和政协民主监督下，依法行政和科学决策的结果；是社会各界和驻军、武警官兵及中省直单位大力支持的结果；也是全市人民励精图治、团结奋斗的结果。在此，我代表市人民政府表示衷心的感谢！

　　在充分肯定成绩的同时，我们也清醒地看到，全市经济和社会发展中还存在一些不容忽视的矛盾和问题。主要是：经济总量小，牵动力强的大项目少，经济发展缺乏后劲；农村经济产业化、市场化程度低，龙头企业少、规模小，带动能力弱；历史积存的机制性、结构性矛盾仍很突出，部分国有企业改革不到位，产业和产品结构调整缺乏深度；对外开放质量和水平不高，利用外资和外贸出口规模不大，临港产业发展缓慢；社会保障压力较大，就业矛盾比较突出，维护社会稳定任务仍很艰巨；财政进入偿债高峰期，加之刚性支出不断增加，收支矛盾进一步加剧；政府职能转变相对滞后，行政效能有待提高，软环境建设尚需加强。这些问题，我们将在今后工作中认真加以解决。

　　二、２００４年工作的主要任务

　　２００４年是实施老工业基地振兴战略的起步之年，也是顺利实现″十五″计划的关键之年，做好今年工作意义十分重大。

　　当前，我们要认真贯彻中央《关于支持东北地区等老工业基地振兴战略的若干意见》，按照中央经济工作会议提出的″东北振兴明年要有良好开端″和省经济工作会议提出的″确保实现高起点快起步开局、高水平跨越式发展″的要求，把思想统一到国家振兴东北等老工业基地的战略部署上来，把力量凝聚到实现营口全面振兴的目标上来。立足我市的区位、资源、产业优势，积极抢抓机遇，全力打造港口经济平台，加速创建全国最大的镁质材料产业基地、服务东北的现代物流基地、领先辽宁的轻纺工业基地，做大做强冶金、石化、装备制造、新型建材和农产品深加工五大产业。牢固树立全面、协调、可持续的发展观，切实把工作重点转移到转变经济增长方式，提高经济效益上来，推动营口老工业基地的全面振兴。

　　２００４年工作总的指导思想是：以邓小平理论和″三个代表″重要思想为指导，全面贯彻党的十六届三中全会精神，按照市委九届七次全会的要求，紧紧围绕老工业基地振兴的总体目标，坚持以解放思想为先导，以结构调整为主线，以改革开放和科技进步为动力，以项目建设为核心，加速建设″一个平台″、″三大基地″、″五大产业″，全力推进新型工业化、农业产业化和服务业现代化，做好社会保障和就业工作，努力实现国民经济持续、快速、协调、健康发展和社会全面进步，为营口老工业基地振兴开好局、起好步。

　　２００４年全市经济和社会发展的主要目标是：国内生产总值３０２．３亿元，比上年增长１８％；固定资产投资１５０亿元，增长４４．８％；外商直接投资３．２２亿美元，增长３０％；外贸出口７．２８亿美元，增长２０％；地方财政一般预算收入１２．３亿元，增长１５％；城市人均可支配收入８０５０元，增长１０％；农民人均纯收入３８４５元，增长８％；城镇登记失业率控制在６％以内。

　　为了实现上述任务和目标，要重点抓好以下十项工作：

　　（一）继续深化经济体制改革，增强经济发展活力

　　要认真贯彻中央《关于完善社会主义市场经济体制若干问题的决定》，把改革贯穿于老工业基地改造的全过程，加速实现体制创新和机制创新，努力在微观上搞好搞活企业，在宏观上创造良好的发展环境。

　　深化国有企业改革，加速建立现代企业制度。要认真贯彻落实国务院《关于规范国有企业改制工作的意见》，以产权制度改革为重点，以股份制改造为主要形式，完善公司法人治理结构，推进企业的机制创新，全年实施重点企业改革４４户。要加快企业分配制度改革，探索技术、管理、资金等各种生产要素参与分配的有效途径，激发经营者和生产者的积极性。强化对特困企业和续存企业的管理，加快处理国有企业遗留问题，剥离企业办社会职能。要规范资产处置行为，通过招商引资、资产变现、土地出让、资产置换及规范破产等形式，盘活资产存量。

　　进一步优化所有制结构，促进非公有制经济健康发展。按照″放心、放开、放手″的原则，不断完善和落实鼓励投资创业的政策，放宽市场准入，强化中介服务。加大金融对非公有制企业发展的支持力度，不断增强担保支持能力。鼓励民间资本参与国有、集体企业改制，扶持有条件的企业做强做大。加强对非公有制企业的服务，充分发挥工商联和各类行业协会的作用。２００４年，非公有制经济实现增加值和实缴税金分别增长２０％以上。

　　进一步深化农村改革，完善农村经济体制。按照″依法、自愿、有偿″的原则，不断完善土地承包经营权流转办法，逐步发展适度规模经营。以民营为主、政府补助相结合的方式，推进水利、林业、农机产权制度改革。继续深化农村税费改革，完善和落实各项政策，做好合乡并镇和教育布局结构调整等后续工作，确保村村减负、户户受益。坚持经营性与服务性相分离的原则，进一步完善农业信息服务体系、农村市场流通体系和农业技术推广体系，发展壮大农村经纪人队伍，强化对农业和农村经济的社会化服务功能。

　　继续推进流通、城建、公用事业等项改革，加速与市场经济接轨。深化流通体制改革，促进国有资本从中小商业企业退出。大力推进粮食购销企业改革，实现粮食收购主体多元化和粮食购销市场化。加快城建公用事业改革，逐步实现基础设施的社会化建设和商品化使用。认真落实上级有关政策，稳步推进事业单位改革。

　　（二）坚持以市场为导向，促进产业结构优化升级

　　振兴老工业基地，必须抓住结构调整这一主线。要按照巩固一产、做强二产、提升三产的原则，进一步优化产业结构，协调发展城乡经济，全年一、二、三产业增加值分别达到３５．８亿元、１６０．６亿元、１０５．９亿元。

　　加快农业产业化和农村城镇化进程，大力发展县域经济。要切实把解决″三农″问题放在突出的位置，不断增加对农业的投入，确保专项资金及时足额到位，进一步巩固农业的基础地位。坚持用工业的理念谋划农业发展，使农业步入以工促农、工农互动的良性发展轨道。按照″稳粮活经、渔牧倍增、果蔬跨越、特产翻番″的思路，将粮经作物比例调整到５５?押４５，订单农业面积达到７５万亩。充分发挥″两水一牧一菜″的优势，加快培育主导产业。提高优质果品比重，水果产量确保４０万吨。加速″海上营口″建设，工厂化养殖面积达到１１万平方米，水产品总产量力争实现３４万吨。加快无规定动物疫病示范区和良种基地建设，扩大绒山羊、肉猪、肉牛、肉鸡生产规模，畜牧业占农业总产值比重达到３５％。加强无公害农产品基地建设，推广标准化生产技术，完善质量安全检验监测体系，提高农产品的市场竞争力。围绕实施公益林保护和退耕还林等项工程，大力发展生态经济型林业，全年人工造林８万亩，植树２６００万株。以水源工程和城市防洪安全为重点，加快水利设施建设。坚持产业拉动、龙头牵动、科技推动和能人带动的原则，促进农业产业化经营，培育壮大龙头企业。全年完成投资７．３亿元，建设农产品加工项目１００个，其中投资千万元以上的２０个。继续支持农业园区的发展，全年完成投资５．６亿元，新上和续建项目８０项。大力发展乡镇企业，全年投入资金４０亿元，开工建设百万元以上项目４６０项，其中千万元以上１３０项。以县城扩容和中心镇建设为重点，围绕改善城镇功能和城镇形象，高起点、高标准地搞好城镇的规划和建设，增强辐射和带动能力。

　　加大产业和产品结构调整力度，做大做强工业经济。振兴老工业基地，重点在工业，难点在工业，希望在工业。要走″工业强市″的发展之路，突出扩大总量，加速企业的技术改造，推进产业和产品结构的整体优化。镁制品生产以提高加工深度为重点，以青花、华晨、金龙等大型镁加工企业为主体，打造镁质材料加工产业集群。冶金行业以建设精品钢材基地为重点，抓好中板轧钢生产线二期配套工程和盼盼４０万吨冷轧板等一批项目；轻工行业以主导产品扩产增效为重点，抓好钢琴、卷烟、冰箱等一批项目；纺织行业以提高产品附加值为重点，抓好熊印织造生产线、华福印染生产线改造等一批项目；石化行业以延伸开发为重点，抓好华晨集团２２０万吨焦化、向阳科化集团１００吨聚丙烯催化剂等一批项目；建材行业以发展新型材料为重点，抓好盼盼建材基地等一批项目。全年完成工业固定资产投资７５亿元；技术改造投资１５亿元。继续实施名牌战略，巩固和发展″诺的斯卡″钢琴、″人民大会堂″卷烟等名牌产品，发挥名牌的带动效应。加强企业管理，不断提高企业的财务、质量、采购和营销管理水平。加快１２个工业园区建设，提高规划和管理水平，发展特色产业和特色产品，拉长和延伸产业产品链条，实现工业园区跨越式发展。全年工业园区固定资产投资、利用外资、规模以上工业产值、实现利税等项指标要高于全市平均增幅２０个百分点以上。

　　提升传统服务业，加速发展新兴第三产业。要按照《营口服务业发展规划》，优化商业网点布局，完善现有商业街、商业区功能。全年计划投资２．８亿元，重点培育和建设家居、机动车等４个专业市场。大力发展连锁经营、物流配送等现代营销方式，推动拍卖、租赁、典当等服务业升级发展。全年实现社会消费品零售额９３亿元，增长１４％。开展对屠宰加工、酒类和粮油产品等特殊行业的综合整治，严厉打击各种制假售假行为，维护消费者权益。积极培育医疗保健、扶老托幼、卫生保洁等社区服务业，实现社区服务产业化、专业化。进一步完善旅游业发展规划，认真搞好旅游项目包装和资源开发，切实加强景区建设与管理，组织好市民韩国游、望儿山母亲节等活动，全年实现旅游总收入１３亿元，增长１４％。

　　（三）深入开展″招商引资年″活动，坚持以开放促调整、促发展

　　要牢牢把握实施老工业基地振兴这一有利契机，增强大开放意识，以开发区为龙头，以招商引资为重点，促进开放型经济发展。

　　坚持内外并举，积极而有效地利用外资。要以项目开发为核心，以建设载体和优化环境为突破口，促进″招商引资年″工作向纵深发展。坚持″五结合、五为主″的原则，即在项目开发上实行内外结合，以外为主；在项目领域上实行生产、建设、流通型相结合，以生产型为主；在项目规模上实行大中小相结合，以大型为主；在项目对接上实行政府、企业相结合，以企业为主；在项目跟踪上实行领导和项目单位相结合，以项目单位为主。全年确保新开工建设投资５００万元以上项目５００项，其中工业项目４００项，利用外资工业项目１００项；新增规模企业２００户；全口径财政收入４０亿元。要本着突出重点、注重实效的原则，围绕建设″一个平台″、″三大基地″、″五大产业″，把韩、日、港、台作为重点，提高招商引资的集中度。积极推进同沿海地区的合资合作，加强同其它发达地区的联系，全年吸引域外资金６０亿元。要改进招商方法，综合运用以商招商、网上招商等形式，不断提高招商引资效率。今年要积极办好韩国、日本和港澳台招商引资活动周，力求取得实效。要加强招商基础工作，依托现有企业搞好项目包装，全年推出重点招商项目５００项。要引导和激励企业充分发挥招商主体作用，推动企业进入项目开发的主战场。要认真搞好项目的筛选和论证，严格履行审批程序，防止盲目投资和低水平重复建设，努力提高投资效益。各级政府和市直有关部门，既要抓好服务和引导，又要组织好项目包装和洽谈，切实进入项目开发角色。要努力创造有利于经济发展和对外开放的政策环境、人文环境和法制环境，进一步提高对投资者、经营者的服务水平。

　　优化出口产品结构，大力开拓国际市场。要积极应对国家出口退税政策的调整，引导出口生产由初加工向深加工转变、由低附加值向高附加值转变，大力发展农副产品和工业制成品出口。今年要重点扶持２５户企业、２２种有市场竞争力的产品扩大出口，保持现有增长势头。要积极培育和壮大外贸出口的主体，支持各外贸专业公司加速发展。

　　充分发挥开发区的龙头作用，增强对外辐射功能。开发区要紧紧围绕亿吨大港建设，充分发挥体制优势、环境优势和基础优势，坚持以利用外资为主、工业项目为主、出口创汇为主，突出港前物流和临港工业发展，集中力量抓好伊斯帕特镀锌板等重大项目的实施，全年吸引外商直接投资１亿美元。加快高新区基础设施建设，强化招商引资和高新技术孵化功能，全年引进工业项目３０个，完成固定资产投资８亿元。

　　（四）坚定不移地实施″以港兴市″战略，努力打造港口经济平台

　　要不断完善港口基础设施，充分发挥港口的辐射带动功能，构筑以港口经济为特色的新型产业体系。

　　加快港口基础设施建设，扩大港口运输能力。重点抓好鲅鱼圈港区三期工程、成品油及液体化工品码头、１０万吨粮食筒仓等一批重点项目建设；积极推进鲅鱼圈港区四期工程、１５万吨级深水航道、港前保税区、沙鲅电气化铁路等重大项目的前期工作，确保全年实现建设投资１３亿元。加强货源基地建设，提高港口服务水平，全年完成港口吞吐量５０００万吨、集装箱运量５５万标箱，分别增长２４．７％、３６．８％。

　　充分发挥港口优势，发展临港工业。要以港口为依托，以招商引资为途径，大力发展以石化、冶金、建材为重点的临港工业。加快辽宁船舶工业园建设，鼓励和支持装备制造业的发展。按照″开发一片、配套一片、建设一片、收效一片″的原则，支持货主码头、临港工业园区的建设，构筑港口经济平台。

　　坚持以港兴贸，培育现代物流业。要充分利用区位和港口优势，有效整合、改造和提升物流资源。大力兴办货运、货代、船代等相关行业和以仓储服务为主的物流业，引进国内外先进的物流企业，建立以贸易、仓储为主的港前贸易区。积极发展工业原材料和零部件采购、产成品配送及仓储运输业，支持民间资本兴办海运事业，促进物流业的生成。

　　（五）加强城镇规划、建设与管理，进一步完善城市总体功能

　　坚持高起点规划、高质量建设、高效能管理、高水平经营的方针，努力改善城乡环境，完善城市功能，全面提升城市化水平。

　　加强市场化运作，继续推进城市经营。按照控制总量、盘活存量的原则，完善国有土地使用权招标、拍卖和挂牌出让制度，规范土地收购、整理和储备工作，全年土地供给控制在６０万平方米左右。加快矿业市场培育，对探矿权、采矿权实行有偿出让。多渠道筹措资金，鼓励非国有资金进入公用设施建设领域。激活房地产二、三级市场，消化处置空置商品房。积极探索城市垃圾处理、供水、供暖、供气、公交等城市资源特许经营权拍卖、有偿使用和经营权转让，盘活城市资产。

　　全力抓好重点项目建设，完善城市基础设施。围绕促进全市经济和社会全面发展，解决关系群众切身利益的热点、难点问题，今年要集中力量为群众办好２０件实事：（１）开工建设滨海大道；（２）改造营大路，实施农村公路网建设；（３）续建盼盼路；（４）改扩建市区部分道路及街巷路灯；（５）改造西市场和牛屯等棚户区；（６）新建城市生活垃圾处理厂、市医疗垃圾处理中心；（７）改造路南河、营柳河潮沟；（８）开工建设市中心医院；（９）新建市传染病院；（１０）扩建市疾病预防控制中心；（１１）新建市高中分校；（１２）新建市消防指挥中心；（１３）建设西部热电厂；（１４）新建南湖公园；（１５）改善环卫设施，新建和改造部分公厕；（１６）更新公交车辆３０台、新增公交线路２条；（１７）易地改扩建市政府机关幼儿园；（１８）续建营口红运广东家居批发商城；（１９）建设新一代天气雷达系统；（２０）新建２２０千伏柳树输变电工程。

　　严格实施城市总体规划，依法推进城市管理。认真贯彻《规划法》，坚决维护规划的权威性和严肃性。抓紧编制市域内城镇规划，科学搞好城市功能分区规划、详细规划和专项规划。加大综合执法力度，逐步形成依法管理、群众参与和科技措施相结合的管理机制。加强社区建设，进一步完善基础设施和服务功能，充分发挥社区在城市管理中的作用。落实门前″五包″责任制，加强市容市貌和道路交通秩序等综合整治。严格执行国家、省物业管理条例，进一步规范物业管理行为，提高小区建设水平。注重城市生态建设，大力实施城市绿化工程，全年市区植树２０万株，新增城市绿地３０万平方米，绿化覆盖率达到３４．４％。进一步治理污水、噪声和大气污染，推进城市垃圾无害化处置，努力提高环境质量。

　　（六）加强财税、金融管理，确保财政收入稳步增长和金融环境明显改善

　　认真实施国家财政、金融政策，坚持开源与节流并重，强化财税、金融工作。

　　抓好财源建设，强化税收征管。充分发挥财政资金的导向作用，采取贴息、垫息、资助等措施吸引资金，支持重点行业、骨干企业和名牌产品发展，努力培植和壮大财源。坚持依法治税，加强税源和税基管理，严格查处串库、串税现象，打击偷、骗、抗税行为，确保税费及时足额入库。

　　创新支出机制，规范支出行为。合理界定公共支出范围，从严控制行政支出，集中财力保证农业、教育、科技、社会保障等重点支出。深化″收支两条线″改革，全面推行部门综合预算、国库集中收付，扩大政府采购范围。强化财政监督，建立健全分配、执行和监督相互分离、相互制约的财政运行机制。深化以″收缴分离″为核心的行政事业性收费管理制度改革，坚决杜绝坐支和私设″小金库″、″帐外帐″等违纪现象。

　　加强债权债务管理，防范和化解财政风险。要规范政府举债、担保行为，建立债务预警机制，严格控制政府举债项目。建立偿债机制，有序推进偿债工作。加强政府债权清收，组织专门力量，制定激励政策，实行依法追缴，尽量减少资金损失。强化国有资产管理，建立国有资产抵押、转让、出售和政府信用贷款的审批监控机制，防止国有资产流失。

　　维护金融安全，拓宽融资渠道。大力整治信用环境，严厉查处金融违法行为，确保不良贷款率比上年下降３个百分点。进一步优化信贷结构，增加贷款投放。加快企业上市步伐，推进盼盼股份上市进程。继续吸收外埠银行对我市的资金投入，创造条件争取域外银行在我市开设机构。

　　（七）以扩大就业和再就业为重点，加速完善社会保障体系

　　做好就业和社会保障工作，是民心工程，也是实践″三个代表″重要思想的根本体现，必须全力以赴抓好。

　　不断拓宽就业渠道，千方百计扩大就业。要把扩大就业放在经济社会更加突出的位置，坚持劳动者自主择业、市场调节就业和政府促进就业的方针，积极发展就业型经济，千方百计创造就业岗位，全年推荐就业５万人。积极开展跨地区劳务协作和劳务输出，全年新建劳务输出基地５个，力争新增境内外劳务输出１万人次。加强对《再就业优惠证》的综合管理，建立健全跟踪监督机制，把鼓励就业的各项优惠政策落到实处。完善就业服务体系，进一步发挥社区劳动保障工作平台的作用。建立和完善培训经费补贴与再就业效果直接挂钩的工作机制，动员全社会力量开展就业培训。适度开发新的公益性岗位，帮助特殊困难群体实现就业。

　　加强社保体系建设，增强社会保障能力。充分运用舆论、行政、法律等手段，扩大社会保险覆盖面，提高基金收缴率。全年基本养老保险参保人数２６．４万人，失业保险参保人数２５万人，基本医疗保险参保人数２７万人；基本养老保险个人帐户记清率、上解率达到１００％；企业离退休人员养老金社会化发放率１００％。全面贯彻《工伤保险条例》，完善工伤保险体系。按照″巩固、规范、提高″的要求，进一步完善城市居民最低生活保障制度，低保金银行发放率达到１００％。积极发展商业保险，充分发挥商业保险在社会保障体系中的作用。

　　构筑长效扶贫机制，保障困难群众的生产和生活。完善扶贫帮困责任制，建立临时救济、包户帮扶、救灾救济与城乡低保相结合的社会救助体系。加强救灾款物管理，实行救灾款物实体下拨、专户存储、封闭运行。加大对困难地区的扶持力度，切实改善生产生活条件。

　　（八）加快发展各项社会事业，促进社会全面进步

　　实施″科教兴市″和″人才强市″战略，协调发展各项事业，为老工业基地振兴创造良好的社会环境。

　　坚持以科技为先导，推动经济发展。采取有效措施，加快信息产业、高新技术产业发展，加速专利技术成果转化，不断提高全市的科技创新能力。全年实施高新技术产业化项目３０项，高新技术产值达到５３亿元，增长２６．２％。抓好农业科技示范和推广，全年引进农业新品种４０个、新技术１０项，完善１３个农业科技示范基地。积极开展厂校协作，全年签订科技协作项目６０项，产学研联合企业达到１９０户，完成技术攻关２０项，完善企业技术研发中心１５个。

　　大力发展教育事业，加速人才和智力开发。以素质教育为方向，深化教育改革，提高教师队伍素质和教学质量。进一步巩固基础教育，大力发展农村教育，高质量、高水平地推进″普九″工作。加快普通高中建设，力争有两所高中进入省级示范性高中行列。积极发展高等教育，做好本科大学筹建工作。大力实施职业教育振兴计划，加大资金投入和教育资源重组力度，优化专业结构，扩大办学规模，为经济发展培养有用人才。坚持积极鼓励、大力扶持、正确引导、依法管理的方针，规范和发展民办教育。加强人力资源管理，推进人才结构调整，创新人才工作机制，优化人才成长环境，培养、吸引和用好各类人才，建设高素质的人才队伍。

　　继续做好文化、卫生、体育、计划生育等项工作，为改革开放和经济建设服务。加强文化基础设施建设，稳步推进文化体制改革，提高文化产品质量。全面深化卫生体制改革，积极实施医疗资源整合，加强农村公共卫生设施建设，建立健全疾病信息网络体系、疾病预防控制体系和医疗救治体系，提高公共卫生服务水平和突发性公共卫生事件应急能力。全面落实″一法三规″和计划生育工作条例，人口出生率和自然增长率分别控制在６．７５‰和２．３‰以内。不断改善广电技术装备水平，努力提高节目编播质量。继续开展群众性体育和竞技体育活动，努力提高全民健康水平。

　　（九）加强社会主义民主法制和精神文明建设，提高依法治市和以德治市水平

　　坚持依法治市与以德治市相结合，建立和完善与社会主义市场经济相适应、与中华民族传统美德相承接的社会主义思想道德体系，促进三个文明协调发展。

　　加强社会主义民主法制建设，全力维护政治安定和社会稳定。进一步完善社会主义民主，自觉接受人大及其常委会的依法监督和政协的民主监督，认真执行人大及其常委会的各项决议、决定，办好人大代表建议和政协提案。加强同各民主党派、工商联、无党派人士和有关人民团体的联系，充分发挥工会、共青团、妇联等群团组织作用，保障人民群众的知情权、参与权和监督权。进一步落实党的民族、宗教和侨务政策，巩固和发展最广泛的爱国统一战线。大力推进社会主义法制建设，深入开展″四五″普法活动，增强全社会的法律意识。强化公安基础建设，强力推进公安警务运行机制和社区警务战略改革。加大社会治安综合治理力度，积极开展专项斗争和重点整治，严厉打击各种刑事犯罪和″法轮功″等邪教组织。切实抓好安全生产，杜绝重特大事故的发生。认真落实信访工作责任制，加强对各类矛盾和纠纷的排查，从源头上、根本上解决重点、难点问题，维护社会的稳定。

　　加强社会主义精神文明建设，努力提高公民的思想道德素质。深入进行党的基本理论、基本路线、基本纲领的宣传教育，用″三个代表″重要思想统领社会主义思想文化阵地。大力提倡″诚信和谐，务实创新″的营口城市精神，积极推广″遵纪守法、团结友善、勤俭自强、敬业奉献″的基本道德规范，着力加强政务诚信、商务诚信、社会诚信建设，全力打造″诚信营口″。增强全民国防意识，加强民兵、预备役部队建设和国防动员、人民防空工作，大力支持军队建设，全力做好″双拥″工作，进一步密切军政、军民关系。

　　（十）认真实践″三个代表″重要思想，努力建设为民、务实、廉洁、高效的政府机关

　　实现老工业基地振兴对政府工作提出了更高的要求，我们必须不断解放思想，创新思维，转变职能，改进作风，努力把政府自身建设提高到一个新的水平。

　　不断解放思想，坚持与时俱进。要紧紧围绕老工业基地振兴这个中心任务，引导全市上下自觉地破除小富即安、满足现状和″等靠要″等思想，进一步增强发展意识、机遇意识和创新意识，凝聚人民群众的力量，放手让一切劳动、知识、技术、管理和资本的活力竞相迸发，促进营口经济在振兴老工业基地的历史机遇中实现跨越式发展。要深化对市情的认识，借鉴先进地区经验，进一步理清和完善振兴老工业基地的思路。要建设学习型政府，做到勤于学习，善于学习，不断适应形势变化和经济发展的需要。

　　加速转变政府职能，不断改善投资环境。认真贯彻《行政许可法》，大力推进″三清理″工作，依法规范政府部门的行政许可行为。深化行政管理体制改革，取消妨碍市场开放和公平竞争的行政审批事项，建立行为规范、运转协调、公正透明、廉洁高效的行政管理体制。严格落实行政执法责任制、评议考核制和行政过错责任追究制，推进依法行政。建立健全政府行为监督制约机制，从源头上制止乱检查、乱罚款、乱收费、乱摊派等影响投资环境的行为。进一步改进调节和监管方式，真正把政府经济管理职能转移到为市场主体服务和创造良好发展环境上来。

　　切实改进工作作风，进一步提高行政效率。加强政府的规范化、制度化和科学化管理，改进领导方式和工作方法，减少不必要的应酬活动，集中精力抓大事、办实事。改进会风和文风，精简会议和文件。坚决反对形式主义、官僚主义和教条主义，大兴调查研究之风，注重解决制约经济发展的突出问题。倡导认真负责、雷厉风行、埋头苦干、求真务实的作风，坚持讲真话、鼓实劲、报实情、求实效，力戒弄虚作假和浮夸之风。加大督查力度，严格考核奖惩，在全市上下形成狠抓落实的良好风气。

　　继续发扬艰苦奋斗的精神，努力做到廉洁从政和为民行政。政府组成人员和全体公务员要继承和发扬党的优良传统，始终保持谦虚谨慎、不骄不躁的作风，始终保持艰苦奋斗、勤俭节约的作风，坚决克服讲排场、比阔气、挥霍浪费、贪图享乐等不良倾向。切实加强党纪、政纪教育，落实廉政建设各项规定，使各级政府公务员增强反腐倡廉的自觉性。加大对违法违纪案件的查处力度，严厉惩处腐败分子，切实纠正损害群众利益的不正之风。坚持防患于未然，建立和完善权、钱、事等方面的管理和监督制度，力求从源头上预防和消除滋生腐败的土壤。要牢记为人民服务的宗旨，做到权为民所用，情为民所系，利为民所谋。要密切联系群众，按照″群众利益无小事″的要求，注重了解民情、反映民意、集中民智、珍惜民力，把心思放在诚心诚意、勤勤恳恳为群众办实事上，努力为群众解决生产、生活中遇到的困难和问题，真正做到执政为民。

　　各位代表，营口老工业基地振兴任重道远，面对新的机遇，新的挑战，让我们在市委的正确领导下，继续高举邓小平理论伟大旗帜，认真实践″三个代表″重要思想，以饱满的热情、昂扬的斗志、创新的精神、扎实的工作，全面完成今年的各项任务，为实现营口的全面振兴而努力奋斗！
